# RegEnrich gene regulator enrichment analysis reveals a key role of the ETS transcription factor family in interferon signaling

**DOI:** 10.1038/s42003-021-02991-5

**Published:** 2022-01-11

**Authors:** Weiyang Tao, Timothy R. D. J. Radstake, Aridaman Pandit

**Affiliations:** 1 Center for Translational Immunology, Department of Immunology, University Medical Center Utrecht, Utrecht University, Utrecht, The Netherlands; 2 Department of Rheumatology and Clinical Immunology, University Medical Center Utrecht, Utrecht University, Utrecht, The Netherlands

**Keywords:** Gene regulatory networks, Machine learning

## Abstract

Changes in a few key transcriptional regulators can lead to different biological states. Extracting the key gene regulators governing a biological state allows us to gain mechanistic insights. Most current tools perform pathway/GO enrichment analysis to identify key genes and regulators but tend to overlook the gene/protein regulatory interactions. Here we present *RegEnrich*, an open-source Bioconductor R package, which combines differential expression analysis, data-driven gene regulatory network inference, enrichment analysis, and gene regulator ranking to identify key regulators using gene/protein expression profiling data. By benchmarking using multiple gene expression datasets of gene silencing studies, we found that *RegEnrich* using the GSEA method to rank the regulators performed the best. Further, *RegEnrich* was applied to 21 publicly available datasets on in vitro interferon-stimulation of different cell types. Collectively, *RegEnrich* can accurately identify key gene regulators from the cells under different biological states, which can be valuable in mechanistically studying cell differentiation, cell response to drug stimulation, disease development, and ultimately drug development.

## Abstract (web summary)

Weiyang Tao et al. describe RegEnrich, an R package capable of conducting several analyses to identify transcriptional regulators driving gene expression signatures. As an example, they apply RegEnrich to dozens of in vitro gene expression datasets to identify that several ETS transcription factor family members are associated with interferon stimulation.

## Introduction

The advances in high throughput technologies such as genomics, transcriptomics, and proteomics have provided unprecedented opportunities to mechanistically understand the genetic and epigenetic alterations in diseases, cellular development, cell stimulation, and immune activation^1,2^. Typically, alterations in the expression of key genes and proteins play a central role in many of these biological states. Thus, to understand the differences between these states, one of the fundamental steps is to identify a set of genes or proteins that are differentially expressed^3^. To understand the underlying biological process and functions of these molecules, annotation enrichment methods, such as pathway and gene ontology (GO) term enrichment, have been used widely^4^. Although the enrichment analysis provides crucial clues about the underlying biological processes and pathways, the lack of information about the underlying regulation hinders us to mechanistically understand how these biological states can be achieved.

To study the function, regulation, and dynamics of individual genes (or proteins) in a complex biological system, network biology is emerging as an important tool^5^. Several studies have demonstrated that constructing gene/protein interaction networks allows us to gain important insights into the regulatory mechanisms that govern different biological states, including disease, cellular activation, and differentiation^6,7^. In a gene/protein interaction network, densely connected genes (or *hub genes*) are crucial for the network’s integrity and the corresponding biological state^6,7^. However, considering only topological parameters (such as hubness or degree) of a network may overlook key regulators^5^. So, to gain regulatory insights, we should consider both network topology and the corresponding alterations in gene or protein expression.

Transcription factors and co-factors (TFs) can directly (and/or indirectly) regulate the expression of multiple target (and/or downstream) genes and proteins^8–10^. Some studies took advantage of curated TF–target networks^11–13^, or predicted network based on ChIP-seq data^14,15^ and used Fisher’s exact test and other enrichment algorithms to identify key/master regulators of the genes of interest^16–18^. However, current curated networks are incomplete, and increasing studies have shown that regulatory interactions may differ over time, upon different conditions and cellular states in the same organism^8,19,20^. So, analyses based on these incomplete static networks might not be sufficient to unveil functional regulatory patterns in complex biological processes.

State/cell/condition-specific gene regulatory network can directly be inferred from the gene or protein expression data (data-driven network)^8,21^. Using these data-driven networks and results from differential expression analyses, one can deduce key regulators. For example, ARACNE and ARACNe-AP, two packages based on ARACNe algorithm, have been used in the reverse engineering field, reconstruct a gene regulatory network from gene expression profile datasets based on mutual information^22,23^. NeTFactor algorithm uses this type of network and applies topological, statistical, and optimization methods to identify key regulators^24^. An R package called VIPER takes advantage of this network and uses *t*-statistics (or other measurements) by comparing gene expression of different conditions to compute the final enrichment *p* values for TFs based on analytic rank-based enrichment analysis (aREA) algorithm and ﻿a null model generated by sample bootstrapping^25^. VIPER has been successful in identifying master regulators in many studies^26–30^. However, it currently utilizes the network reverse-engineered by ARACNe that typically requires a large number of samples, which may not always be fulfilled, to successfully build a robust network, thus hampering VIPER’s broader application.

**Fig. 1:**
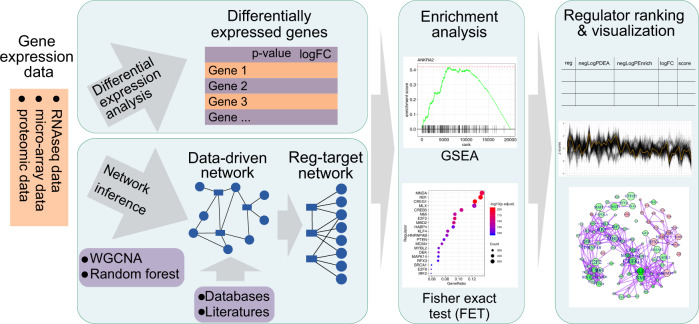
The analytic workflow of *RegEnrich* package. *RegEnrich* consists of four major steps: differential expression analysis, regulator-target network construction, enrichment analysis, and regulator ranking and visualization.

Here, we developed “*RegEnrich*”, an open-source R package for gene regulator enrichment analysis (Fig. 1). The *RegEnrich* pipeline aims to identify the key regulators based on their differential expression and enrichment of their potential downstream targets from a given gene set. Because the gene regulators do not act alone but function as part of a complex network, by using *RegEnrich*, one can refine a key gene regulatory network to study the biological process and visualize the derived network.

## Results

### Time consumption and memory usage by *RegEnrich*

**Fig. 2:**
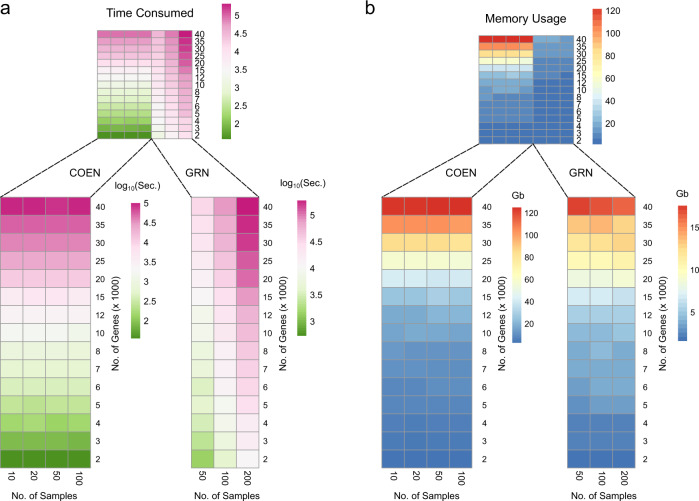
Time consumption and memory usage by *RegEnrich*. With one CPU core, (**a**) the time consumed and (**b**) maximum memory used by *RegEnrich* when analyzing a gene expression dataset with different numbers of genes (ranging from 2000 to 40,000) and different numbers of samples (ranging from 10 to 100 and from 50 to 200 for COEN and GRN network, respectively).

The most time- and memory-consuming procedure of the *RegEnrich* pipeline is inferencing a regulator-target network from the gene expression data. Here, we benchmarked the time consumption and memory usage of different methods in the *RegEnrich* package using Intel^®^ Xeon^®^ Processors with 1, 4, 8, and 16 cores on CentOS Linux 7 operating system on high-performance computing facility at University Medical Center Utrecht (Fig. 2 and Supplementary Fig. 1). And the gene expression data were simulated with different numbers of samples (10, 20, 50, 100 for the COEN method and 50, 100, 200 for the random forest method (GRN)) and different numbers of genes (from 2000 to 40,000). Overall, the speed of both methods decreased with the increase of the number of genes, and the speed was also dependent on the sample size for only the GRN method (Fig. 2a). More specifically, the consumed time of the COEN method increased quadratically with the number of genes, while independent of the sample size. The COEN method was around 1 ~ 100 times faster, compared to the GRN method, when the number of genes was below 20,000, and the number of samples was over 50. However, since the GRN method is linearly, rather than quadratically, dependent on the number of genes, The COEN method spent more time when the number of genes was above 25,000 and the sample size was below 100. Network construction using the GRN method running on 4, 8, and 16 CPU cores was on average about 2, 4, and 8 times faster than the single-threading implementation, respectively (Supplementary Data 1 and Supplementary Fig. 1).

The maximum memory usage of the COEN method again increased quadratically with the increase of the number of genes and independent with the number of samples (Fig. 2b). Given the same size of the simulated expression data, the memory used by the COEN method was more than that by the GRN method in almost all circumstances. In general, using a dataset of 50 samples based on 1 CPU core, when constructing a protein-coding network, which comprises about 2 × 10^4^ genes, it costs ~3.4 h (38.5 Gb) and ~4.1 h (8.2 Gb) by the COEN and by the GRN method, respectively. And it costs 1.6 h (7.8 Gb) by the GRN method using 4 CPU cores. The figure may be different depending on the computing power of the processors, but the order of magnitude will less likely change. So, the users can roughly expect the time and memory usage when performing the *RegEnrich* analysis according to Fig. 2 and Supplementary Fig. 1 and Supplementary Data 1.

### Comparisons of key regulators obtained by different methods

Increasing studies predict key gene regulators by the hubs in a network, which is defined by topological features, such as degree and closeness centrality^31,32^. The degree of a node is the total number of nodes connected to this node in a network. The out-degree of a node is the number of nodes pointed by this node in a directed network. The closeness of a node is defined as the reciprocal of the sum of the shortest path length between this node and all other nodes in the network^33^. And the out-closeness of a node is defined as the reciprocal of the sum of the shortest path length from this node to all other nodes in a directed network.

**Fig. 3:**
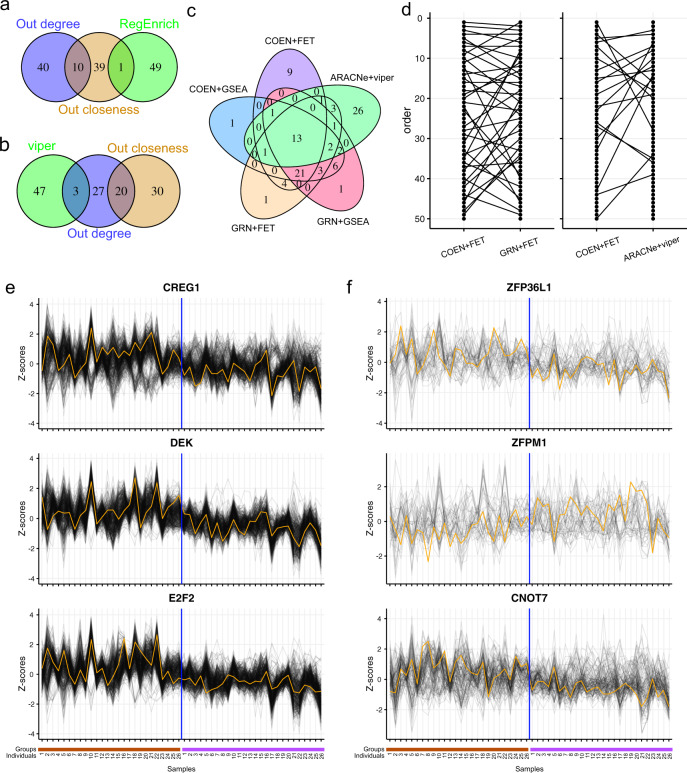
Comparison of key regulators (or hubs) identified by different methods. Venn diagram shows the overlap between the top 50 hubs/regulators **(a)** defined by out-degree, out-closeness and the *RegEnrich* score (using COEN network and FET enrichment method), **(b)** defined by out-degree, out-closeness and the VIPER package (using ARACNe network). **(c)** Venn diagram shows the overlap between the top 50 hubs/regulators defined by *RegEnrich* (using different parameter combinations) and those by the VIPER package. **(d)** Ladder plots compare the rank of regulators identified by *RegEnrich* using different network inferencing methods, and those by *RegEnrich* and by VIPER. Lines connect the same regulators. The expression pattern of top three key regulators and their targets identified by **(e)** *RegEnrich* using COEN network and FET method and by **(f)** VIPER. Orange lines are the normalized expression of regulators and grey lines are the normalized expression of the targets of the regulators. The brown bars below the x-axis indicate samples at week 1, and purple bars the samples at week 3. The analyses were performed using the data obtained from^34^.

We first checked how many key/master regulators identified by the *RegEnrich* package are hubs defined by the network topological properties. We downloaded a publicly available RNA-sequencing transcriptomic dataset, a total of 97 samples, from the Gene Expression Omnibus (GEO) database^34^. In that study, peripheral blood mononuclear cell (PBMC) samples from 29 patients with acute Lyme disease and 13 healthy controls were used to investigate longitudinal changes of the transcriptomes from the time of diagnosis (V1) to immediately after the completion of a 3-week course of doxycycline treatment (V2), and to 6 months after the completion of treatment (V5)^34^. Here, our interest is to investigate which regulators played the important role in regulating the transcriptomic changes of patients between V1 and V2. Thus, we retained 26 paired RNA-seq samples (three are removed because of quality control according to^34^) on V1 and V2 for RegEnrich analysis. And the network hubs are identified by ranking out-degree and out-closeness of the network generated by RegEnrich. As a result, we obtained three sets of hubs defined by 50 regulators with the highest out-degree (degree hubs), out-closeness (closeness hubs), or *RegEnrich* ranking score (*RegEnrich* key regulators). We found that 20% (10) of regulators were overlapping between degree hubs and closeness hubs, while *RegEnrich* key regulators were barely overlapping with degree hubs or closeness hubs (Fig. 3a).

Given the successful applications of the VIPER package in identifying master regulators in studying many biological questions^26–30^, we subsequently checked how many master regulators identified by the VIPER package are network hubs. A gene regulatory network is built based on those 26 paired RNAseq samples by the ARACNE-AP package and we applied out-degree, out-closeness, and the VIPER package to identify hubs (and master regulators) in this network. Similarly, a little more overlapping regulators were observed between degree hubs and closeness hubs in the network constructed using the ARACNE-AP package, and these hubs are also barely the key regulators identified by the VIPER package (Fig. 3b). Different network-inferencing methods and enrichment methods within *RegEnrich* showed consistent top-ranked regulators (Fig. 3c, d), in which ~40% were also identified by VIPER (Fig. 3c). Altogether, both *RegEnrich* and VIPER tend to rank the nonhub regulators to be the key regulators. Using the network reverse-engineered from the full dataset of all 97 samples in^34^ did not show the exactly the same results but still ranked the nonhub regulators to be the key regulators (Supplementary Fig. 2). These results suggest that looking for a gene/regulator with a high degree or other centrality parameters may not be ideal for identifying key gene regulators in every biological process.

Although ~40% of regulators were commonly ranked in the top 50 regulators by both *RegEnrich* and VIPER, most of the top 10 regulators identified by these two packages are unique (Fig. 3d). Clearly, the expression of the three topmost-ranked regulators (*CREG1*, *DEK*, and *E2F2*) identified by *RegEnrich* was differential between V1 and V2 and showed a very similar pattern to that of corresponding targets (Fig. 3e and Supplementary Fig. 3). Although the expression of three topmost-ranked regulators identified by VIPER was also differential, only the expression of the targets of *ZFP36L1* and *CNOT7* was modestly correlated with that of the regulators themselves (Fig. 3f). One may argue that it is inevitable that the expression of a regulator and its targets are highly correlated in COEN networks and this correlation may not hold for the network that was built by ARACNE-AP using mutual information. However, such correlations between the regulator and its targets were still observed when the network is built using random forest, which is also a nonlinear method (Supplementary Figs. 3 and 4). Thus, *RegEnrich* can successfully, as intended, identify the key regulators that both their expression and their targets’ expression associate with the biological process of interest.

### *RegEnrich* can identify the key regulators in gene-silencing studies

RNA interference (RNAi)-mediated gene-silencing has been widely used to study the biological function of the silenced gene. In a gene regulator silencing experiment, the successfully silenced gene is typically the key regulator that has been malfunctioned. The performance metrics of *RegEnrich* and VIPER were compared by the ability to rank the silenced gene as one of the top key regulators. Here nine gene-silencing experiments from four independent datasets^16,17,35,36^ were used to benchmark *RegEnrich* using COEN network with either FET or GSEA enrichment methods, and VIPER using the network built by the ARACNE-AP package. Multiple cell lines/types, different silenced genes, and varied numbers of samples were deliberately included in these datasets to evaluate the bias induced by these variables (Table 1). *RegEnrich* with either FET or GSEA enrichment method outperformed VIPER in all these datasets, and within *RegEnrich*, GSEA outperformed FET in most cases. For example, using the GSEA method, *STAT3* and *FOXM1* were ranked as the top key regulators when *STAT3* and *FOXM1* were silenced in the BTIC and *ST486* cell lines, respectively (Table 1). *STAT3* and *FOXM1* were also ranked high (the second key regulators) in these experiments when we applied *RegEnrich* with the FET method. Interestingly, in the GSE17172 dataset, although *FOXM1* was not ranked as the first regulator using *RegEnrich* FET method, other two genes (*FOXN3* and *FOXG1*) from the same FOX transcription factor family were ranked as the first and fourth regulator, respectively (Supplementary Table 1). This implies that at least several members of the FOX family were perturbed by *FOXM1* silencing due to either off-targeting or downstream transcriptional signaling and can be inferred by *RegEnrich*. It may also attribute to the “pleiotropy of regulators”^25^ or “shadow effect”^17^, meaning that some of the transcriptional targets of *FOXM1* are also part of the regulons of other FOX family members. By applying VIPER to this dataset, *STAT3* was ranked as the 7^th^ regulator. However, *FOXM1* was failed to be identified by using VIPER maybe because the sample size is small (Table 1).

**Table 1:** The transcription regulators identified by *RegEnrich* and VIPER in gene-silencing studies^*^.

GEO accession	Silencing technology	No. of samples	Cell line	Silenced gene(s)	Ranking		
					RegEnrich (FET)	RegEnrich (GSEA)	ARACNE + VIPER
GSE19114^16^	shRNA	44	BTIC	STAT3	2	1	7
GSE17172^17^	shRNA	9	ST486	FOXM1	2	1	–
GSE19114^16^	shRNA	12	SNB19	STAT3	14	5	n/a
GSE2350^65^	siRNA	8	Burkitt lymphoma cell line	BCL6	32	11	–
GSE19114^16^	shRNA	12	SNB19	CEBPB	50	24	n/a
GSE19114^16^	shRNA	44	BTIC	STAT3 & CEBPB	2 & 285	1 & 957	6 & n/a
GSE19114^16^	shRNA	12	SNB19	STAT3 & CEBPB	365 & 6	38 & 11	n/a & n/a
GSE51978^35^	shRNA	9	IMR32	CHAF1A	10 & 29^#^	15 & 46^#^	–
GSE19114^16^	shRNA	44	BTIC	CEBPB	913	793	n/a

^*^ “n/a” means no result for the regulators of interest obtained after ranking procedure. “–” indicates that ARACNE failed to construct a network based on the dataset. ^“#”^means the ranking on day 5 and day 10 according to the experimental setting.

Similarly, *STAT3* and *CEBPB* in SNB19 cell line, *CHAF1A* in IMR32 cell line, and *BCL6* in Burkitt lymphoma cell line were identified by *RegEnrich*, with the GSEA method, as one of the top 20 key regulators in each corresponding dataset. The rankings of these regulators were considered high because these were regulators popping up from a total list of 1712 regulators in these *RegEnrich* analyses. Meanwhile, we also assessed the *RegEnrich* using two datasets, where *STAT3* and *CEBPB* were tried to be simultaneously silenced in either BTIC or SNB19 cell lines. Even though these two genes were intended to be silenced, only one gene was successfully silenced. More specifically, *STAT3* but not *CEBPB* was successfully silenced in the BTIC cell line, and *CEBPB* but not *STAT3* was successfully silenced in the SNB19 cell line. Thus, only *STAT3* and *CEBPB* were expected to be top-ranked as key regulators in the BTIC cell line and SNB19 cell line, respectively, which were the results returned by *RegEnrich* (Table 1). To evaluate *RegEnrich*’s ability to filter the false-positive results, we included a dataset where *CEBPB* was not successfully silenced in the BTIC cell line. All three approaches did not rank *CEBPB* as one of the top regulators (Table 1).

Since the ARACNe algorithm needs a large sample size to reverse-engineer a robust gene regulation network, and the sample sizes of the datasets here are small, we then used the publicly available network to perform master regulator inference with the viper package (see supplementary methods). *RegEnrich* (GSEA) out-performed the VIPER algorithm except in one of the experiments (Table 1 and Supplementary Table 2). Altogether, *RegEnrich* with COEN network and GSEA method is robust to identify the key regulators in well-controlled in vitro experiments even when the sample size is small.

### *RegEnrich* retrieves interferon related regulators

In human, there are three types of interferons (IFN): type I IFNs (IFNα, β, ε, κ, and ω); type II IFN (only IFNγ); and type III IFNs (IFNλ1, λ2, λ3, and λ4)^37,38^. Due to the great therapeutic value of IFNs against virus infection and cancer, multiple studies have been performed to study the regulatory mechanisms of IFNs and interferon-stimulated genes (ISGs). For example, it has been revealed that extracellular IFNs activate cells by a signal transduction cascade, including activating transcription factors STATs and/or IRFs, leading to the induction of hundreds of ISGs, and forming a frontline of defense against virus infections^37,38^. However, the mechanisms underlying the regulation of most of these ISGs may vary between different cell types and tissues and remain incompletely understood.

Given the potential of identification of key regulators by *RegEnrich* in a biological process, we sought to identify the key regulators by which IFNs stimulated cells to express ISGs. We retrieved and analyzed 11 microarray or RNAseq datasets from the GEO database, comprising 21 in vitro experiments, in which different cells were stimulated by either type I or type II IFN (Table 2). We found that *RegEnrich* identified STAT transcription factor family members, including *STAT1* and *STAT2*, in most IFN stimulation experiments, which is supported by the well-known IFN signaling pathway^38^. In addition, IRF (interferon regulatory factors) transcription factor family members, such as *IRF9* and/or *IRF7*, were also identified as key regulators in a majority of the type I IFN stimulation experiments (Table 2). These IRFs have been reported to play important roles in producing type I IFN downstream receptors that detect viral RNA and DNA, and in regulating interferon-driven gene expression^39^.

**Table 2:** The transcription factors identified by *RegEnrich* in interferon stimulation studies^*^.

Type	Interferon	Time	Concentration	Cell type/line	Families of transcription factors			GEO accession
					STAT	IRF	ETS	
Type I	IFNa	6 h and 12 h	500U/ml	HT1080	STAT1 (1), STAT2 (6), STAT5B (22)		ELF1 (14)	GSE31019^66^
		6 h	500U/ml	SKOV3	STAT1 (2), STAT2 (11)	IRF7 (5), IRF1 (13), IRF2 (18)	ETV6 (20), ELF1 (30)	GSE31019^66^
		6 h	10 U/mL	Primary Hepatocytes	STAT2 (12), STAT1 (24)	IRF7 (4), IRF9 (7), IRF1 (17), IRF6 (29)	ETV7 (1), ETV6 (27), ELK4 (28)	GSE31193^67^
		24 h	10 U/mL	Primary Hepatocytes	STAT2 (1), STAT4 (7), STAT1 (9)	IRF7 (3), IRF9 (11)	ETV7 (4), ELK4 (17)	GSE31193^67^
		10 h	1000 U/ml	Fibroblast	STAT1 (8), STAT2 (24)	IRF9 (7)	ELF1 (32)	GSE67737^68^
	IFNa2	6 h	1000 U/ml	Keratinocyte	STAT1 (6), STAT2 (9), STAT5A (32)	IRF7 (20), IRF1 (21), IRF6 (25), IRF2 (28)	ETV7 (11), ELF1 (26)	GSE124939^69^
		18 h	1000 U/ml	Primary macrophage	STAT3 (9), STAT1 (13)	IRF1 (26), IRF7 (27)		GSE30536^70^
	IFNa2b	2 h	1000 U/ml	EBV-transformed B lymphoblastoid cell lines			ELF1 (2)	GSE117637^71^
		2 h	1000 U/ml	Fibroblast		IRF2 (6)	FLI1 (29)	GSE117637^71^
	IFNa6	6 h	1000 U/ml	Keratinocyte	STAT1 (14), STAT2 (17)	IRF7 (11), IRF1 (28), IRF6 (30)	ETV7 (9), ELF1 (32)	GSE124939^69^
	IFNb	6 h	1000 U/ml	Keratinocyte	STAT1 (8), STAT2 (15)	IRF1 (5), IRF7 (6), IRF2 (19), IRF6 (20)	ETV7 (1), ELF1 (18)	GSE124939^69^
		10 h	1000 U/ml	Fibroblast	STAT1 (10), STAT2 (30)	IRF9 (8)	ETV7 (34), ELF1 (35)	GSE67737^68^
Type II	IFNg	6 h	5 ng/ml	Keratinocyte	STAT3 (13), STAT1 (23), STAT2 (24)	IRF2 (1), IRF1 (5)	ETV7 (8)	GSE124939^69^
		20 h	10 ng/ml	Monocyte-derived macrophages	STAT1 (2), STAT2 (25)	IRF1 (1), IRF9 (31)	ETV7 (3)	GSE79077^72^
		6 h	20 ng/mL	Monocytes	STAT2 (9), STAT1 (11)	IRF1 (3), IRF9 (15), IRF7 (22)	ETV7 (1)	GSE36537^73^
		18 h	20 ng/mL	Monocytes				GSE36537^73^
		18 h	20 ng/mL	Monocyte-derived macrophages	STAT1 (17), STAT2 (34)	IRF1 (2), IRF7 (35)	ETV7 (1)	GSE36537^73^
		24 h	20 ng/mL	keratinocytes	STAT3 (3)	IRF1 (1), IRF2 (10), IRF7 (14)	ETV7 (2)	GSE12109^74^
		24 h	100 U/ml	Peripheral blood derived macrophages	STAT3 (4)	IRF7 (3), IRF1 (8), IRF9 (26)	ETV7 (2), ELF4 (27)	GSE11886^75^
		10 h	1000 U/ml	dermal fibroblast	STAT1 (8), STAT3 (23)	IRF1 (4), IRF9 (10)	ETV7 (11)	GSE67737^68^
		3 h	n.a.	Monocyte-derived macrophages	STAT2 (12), STAT6 (30)		ETS2 (5), ELK1 (24)	GSE130567^76^

^*^Three families of transcription factors were assessed for 21 datasets, where cells were stimulated by different interferons. Only TFs in STAT, IRF, and ETS family ranked in top 35 were shown in “Families of transcription factors” columns as a format of “Regulator (ranking)”. The best combination of parameters (i.e., COEN network and GSEA enrichment method) identified in Table 1 was used in the analysis.

Recent work has shown that *ELF1* (a member of the ETS transcription factor family) is induced by IFN, but does not feed-forward to induce interferons, and transcriptionally programs cells with potent antiviral activity^40^. Interestingly, *ELF1* was identified by *RegEnrich* as one of the key regulators in most of the type I IFN stimulation experiments (Table 2). We further investigated whether any other members of the ETS transcription factor family were also identified by *RegEnrich*. Interestingly, we found another ETS transcription factor family member, *ETV7*, in the lists of top regulators from more than half of type I IFN stimulation experiments and from almost all type II IFN stimulation experiments. A more recent study showed that *ETV7* preferentially targeted a subset of antiviral ISGs crucial for IFN-mediated control of viruses, such as influenza and SARS-CoV-2^41^.

**Fig. 4:**
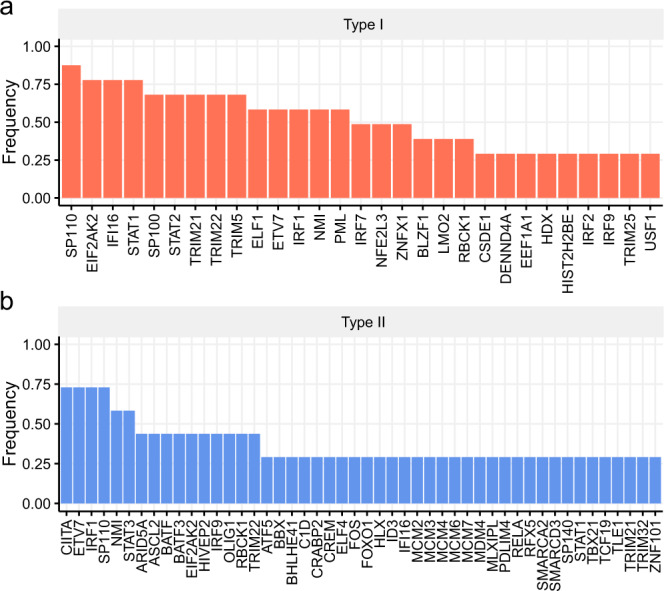
The genes consistently identified as key regulators. Key regulators in **(a)** type I interferon stimulation and **(b)** type II interferon stimulation datasets. The full list has been shown in Table 2. The top 35 regulators in each dataset were included as key regulators, and only the regulators identified in more than 25% of datasets were shown.

Different cells may respond differently to IFN stimulation with different durations. We further investigated the common regulators involved in IFN stimulation among different cells. Thus, we summarized the most common regulators within type I and type II IFN stimulation experiments. It showed that the ISGs of type I IFNs were strongly regulated by STAT family, TRIM family, IRF family, ETS family, SP100/SP140 family (transcriptional coactivator of ETS family TFs). Similarly, type II IFN ISGs were largely regulated by STAT family, IRF family, ETS family, MCM family, SP100/SP140 family (Table 2 and Fig. 4). One of the most commonly identified regulators of type II IFN ISGs was the MHC class II transactivator (*CIITA*), which has been very recently shown with the potential to induce cell resistance to the Ebola virus and SARS-CoV-2^42^. Altogether, these results suggest that *RegEnrich* successfully identified key regulators related to IFN signaling in IFN stimulation experiments.

## Discussion

High throughput technologies like microarray, RNA-seq, and protein mass spectrometry offer easy, fast, and affordable profiling of the gene/protein expression. These technologies generate massive data facilitating us to study the alterations in gene/protein expression, thereby helping us identify the biomarkers for diseases and biological states. However, it is still challenging to predict which genes play major roles in these biological contexts. To address this problem, we developed *RegEnrich*, an open-source R/Bioconductor package integrating differential gene expression analysis, network inference, enrichment analysis, and regulator ranking. *RegEnrich* is able to identify the key regulators by providing gene/protein expression data from multiple high throughput technologies. We benchmarked the speed and maximum memory usage of network inference methods in the *RegEnrich*, which shows that the COEN method runs much faster than the random forest method does when the number of genes is below 20,000, and the speed of the multithreaded random forest version is significantly improved. Traditionally, COEN is considered a method for depicting linear relations between genes, while the random forest for nonlinear relations. Strikingly, in the Lyme disease transcriptomics dataset, the results from the COEN and GRN methods were consistent. This might be because the COEN methods re-evaluate the edge weights by considering the information of neighbor nodes; as a result, such a network was constructed not only based on a linear relationship.

Since hub nodes have been found to be important in many networks, hub genes that are defined by gene regulatory network properties are also expected to be crucial in biology and have drawn much attention over the last decades^43^. We compared the hubs identified by the network properties, i.e., degree and closeness, with the key regulators by *RegEnrich*. Interestingly, only a very small number of the key regulators from either *RegEnrich* or VIPER were hubs (Fig. 3a, b and Supplementary Fig. 2). One possible reason is that these hub genes are so important in maintaining the major functions of cells that too strong perturbations of these hubs could be fatal for cells^44^. Therefore, these hub genes are not necessarily the key gene expression regulators in a specific context. For example, Gaiteri et al. showed that differentially expressed genes primarily reside on the periphery of co-expression networks for neuropsychiatric disorders such as depression, schizophrenia, and bipolar disorder^45^.

As one easy-to-use software/package whose functionality was similar to *RegEnrich*’s, the VIPER R package was used to compare with *RegEnrich* to find the final key regulators. According to the VIPER package instruction on Bioconductor, VIPER needs an ARACNE network to perform the analysis. Such network is generated by an independent package, such as *minet*^46^, *GPU-ARACNE*^47^, and *ARACNe-AP*^23^. These packages either are not R packages or fail to construct the network when the sample size is small. In contrast, *RegEnrich* is an all-in-one package, and a detailed tutorial document is provided along with *RegEnrich*, which facilitates users to use it more easily. More importantly, *RegEnrich* can identify the key regulators whose expression and targets’ expression correlate with the experimental phenotypes. In addition, *RegEnrich* is able to find key regulators not only between two conditions but also in a time series experimental setting. Since *RegEnrich* is modular and is intended to be a flexible pipeline, we allow users to provide custom regulator lists and to have options for multiple methods at different steps in case needed.

Since the datasets in the benchmarking analyses were of a small sample size. We asked whether the sample size influences the performance of the RegEnrich pipeline and the VIPER algorithm. To answer this question, we generated a dataset comprising of *N* samples (*N* = 100), simulating the gene expression of two cell states. These samples were evenly assigned into two groups. We first assumed that all samples had m common gene regulators (*m* = 500), of which *k* were bona fide key regulators (*k* = 20). The expression of each bona fide key regulator followed a Gaussian distribution in each group and was on purpose simulated to be differential between two groups. While the expression of the other regulators followed a Gaussian distribution with the same mean and standard deviation in each group, thus not necessarily differential between the two groups. Then we assumed that each regulator had t targets (t is a random number ranging from 3 to 50). The expression of the targets of a certain regulator was dependent on the expression of this regulator (the expression of this regulator plus Gaussian-distributed values). This resulted in an expression dataset with M genes (*M* = 13,789) and N samples (*N* = 100). Subsequently, we reduced the sample size by a down-sampling strategy, resulting in datasets of 50, 20, 10, and 6 samples. Based on these datasets, we checked whether those bona fide key regulators that we predefined were top-ranked by the *RegEnrich* pipeline. The results showed that with the decrease of the sample size, less bona fide key regulators were top-ranked (Supplementary Fig. 5), meaning that the performance of the *RegEnrich* pipeline could be improved by increasing the sample size. As expected, the random forest (GRN) method in *RegEnrich* and ARACNe failed to build a network when the sample size was smaller than 20. Next, we checked whether the performance would be increased when we used the network that was built based on the complete dataset (all 100 samples) (Supplementary Fig. 6). Indeed, all methods showed a higher performance. Although we observed that the ARACNe + VIPER packages showed a slightly better performance when the sample size was smaller than 20, the network was built based on the full dataset of 100 samples, which is an ideal scenario and hard to achieve in reality. Thus, we believe that RegEnrich is important especially in the area where not so much gene expression data are available.

The regulation of genes is a very complex process, in which many aspects affect the expression of a gene, such as the accessibility of chromatin, single nucleotide polymorphisms, DNA modifications, histone modifications, the expression of its upstream regulators, RNA degradation, and post-translational modifications. Thus, as one of the major steps of the *RegEnrich* pipeline, assuming the key regulators to be differentially expressed between different biological states may not hold in all circumstances. RegEnrich would be failed to predict those regulators whose post-translational modifications but not gene expression changes regulate their downstream gene expression. In addition, an accurate gene regulatory network is very important in identifying the key regulators^25^. RegEnrich inferred the data-driven network by only gene/protein expression data by default. Glass et al. have shown that integration of protein-protein interaction, protein-gene interaction, and gene expression can increase the accuracy of regulatory network inference^48^. Currently, we provide an option for the users to provide their gene regulatory network, which can be derived from other epigenetic datasets such as ChIP-seq, ATAC-seq data, protein-protein interactions, etc., thus, granting *RegEnrich* an ability to integrate multi-omic data.

By analyzing the datasets of dozens of IFN-stimulation experiments, *RegEnrich* identified STAT and IRF transcription factor family members, including *STAT1*, *STAT2*, *IRF9*, and *IRF7*, which have been extensively shown to play important roles in IFN signaling pathways^38,39^. Meanwhile, *RegEnrich* also identified several ETS transcription factor family members, such as *ELF1* and *ETV7*, as key regulators in IFN signaling. Interestingly, *ELF1* transcriptionally program cells with potent antiviral activity and *ETV7* targeted antiviral ISGs crucial for IFN-mediated control of viruses, including influenza and SARS-CoV-2^40,41^. These antiviral activities are typically the fundamental role of IFN in innate immunity. By analyzing the most commonly top-ranked regulators, *RegEnrich* predicted a list of candidate key regulators, such as *CIITA* and *SP100*/*SP140* family members. Given that *CIITA* has been recently reported with antivirus ability^42^, further study may be carried out to investigate the antivirus potential of *SP100*/*SP140* family members, such as *SP100* and *SP110*, which might facilitate the mechanistic studies of IFN-ISG signaling and ultimately drug development.

Recently, using the RegEnrich pipeline, we predicted a network of key regulators that leads monocyte-derived dendritic cells (moDCs) to differentiate into a different trajectory upon CXCL4 stimulation compared to the moDCs without CXCL4 stimulation. We also experimentally validated the *RegEnrich* pipeline’s prediction by silencing one of the top-ranked regulators in the predicted network, i.e., *CIITA*^49^. More recently, we studied the mechanism of human T regulatory (Treg) cell programming under inflammatory conditions. Using RegEnrich, we predicted a network of key regulators important for effector Treg differentiation, including the vitamin D receptor (*VDR*), which is further validated by H3K27ac and H3K4me1 ChIP-seq experiments^50^. These two independent experimental studies support that *RegEnrich* is able to accurately rank the key gene regulators that are mechanistically involved in immune cell development and functions.

Understanding the key regulators between different biological states is essential for gaining mechanistic insights, designing functional experiments, and rational drug development. To this end, here, we presented *RegEnrich*, a Bioconductor R package for inference of key regulators in biological conditions. There are four major steps to obtain the list of key regulators in *RegEnrich*, i.e., differential expression analysis, regulator-target network inference, enrichment analysis, and regulator ranking. For differential expression analysis, the methods in DESeq2 and limma packages are provided, which grants *RegEnrich* the ability to predict the key regulators not only for gene expression data of two conditions but also for time series data. Meanwhile, two regulator-target network inference methods (WGCNA and random forest) are provided, which allows the network to not only contain the linear information but also include a nonlinear relationship between genes. FET and GSEA algorithms are optional for users to perform enrichment analysis. *RegEnrich* can identify the key regulators whose expression and their targets’ expression correlate with the experimental phenotypes. Using datasets from gene-silencing studies, *RegEnrich* using the GSEA method performed the best to retrieve the key regulators and outperformed the VIPER package. Further, by analyzing dozens of in vitro interferon-stimulation gene expression datasets, *RegEnrich* revealed that not only IRF and STAT transcription factor families played an important role in cells responding to IFN but also several ETS transcription factor family members, such as *ELF1* and *ETV7*, were highly associated with IFN stimulations. Above all, *RegEnrich* can accurately identify, in a data-driven manner, key gene regulators from the cells under different biological states, which can be valuable in mechanistic studies of cell differentiation, cell response on drug stimulation, and disease development, ultimately in drug development.

## Methods

The *RegEnrich* is a modular pipeline and consists of four major steps: (a) differential expression analysis; (b) regulator-target network construction; (c) enrichment analysis; and (d) regulator ranking and visualization.

### Differential expression analysis

*RegEnrich* pipeline can be applied to multiple gene expression datasets, including RNA sequencing (RNAseq), microarray, and proteomic data. The first step of finding the key regulators is to obtain differentially expressed genes or proteins (DEs), corresponding differential significance *p* values (${P}_{D}$), and fold changes between conditions. Concerning two-group comparison, here, *RegEnrich* incorporates the Wald significance test from the DESeq2 package and the empirical Bayes method-based linear modeling from limma package to perform the differential expression analysis on RNAseq data and microarray/proteomic data, respectively^51,52^. Regarding the comparisons in experiments with multiple groups or more complex scenarios such as time-series study, the negative binomial generalized linear model-based likelihood ratio test from DESeq2 package^51^ and linear model-based likelihood ratio test are implemented for RNAseq data and microarray/proteomic data, respectively.

### Regulator-target network inference

There are two major types of gene regulatory networks (or regulator-target networks) proposed: static network and dynamic network^53–55^. In a static network, genes are expressed in a steady state thus cannot describe the dynamics of an evolving process, while genes are dynamical in a dynamic network^56^. These networks can be constructed by many different computational approaches^21,22,57–60^. Here, the regulator-target network inference is based on four assumptions: (1) the gene regulatory network is a snapshot of a dynamic network within the users’ experiments; (2) It is a directed network, where the edges are from a regulator to its targets, or from a regulator to its targeted regulators; (3) the potential regulators are transcription factors and co-factors (this can be changed in *RegEnrich* by users); and (4) the expression change of a regulator can lead to the expression change of its downstream targets. Here, the targets are not only direct targets that the regulator binds to but also the downstream genes whose expression can be perturbed by the regulator. Presently, *RegEnrich* provides users two basic options to infer regulator-target network, i.e., COEN (co-expression network) and GRN (based on the random forest algorithm).

For COEN, here, the co-expression network is constructed using WGCNA (weighted gene co-expression network analysis) algorithm^58^. And it can be summarized as three major procedures. First, a similarity matrix is calculated using correlations in expression data to measure the relationship strength between each pair of genes (nodes). Second, by applying the approximate scale-free topology criterion, raising the co-expression similarity to a power to define the weighted network adjacency matrix. Third, this adjacency matrix is then used to calculate the topological overlap measure (TOM), which reflects not only the similarity of each pair of nodes but also their neighbors’ similarity^6^. The TOM defines the final co-expression network^58^.

For GRN, this ensemble regression tree-based method was initially described in GENIE3, which was the best performer in the DREAM4 *In Silico Multifactorial* challenge^21^. The basic idea of GENIE3 is that each gene is regressed in turn against all other genes to obtain network weights (edge weights), which quantify the strength of the dependence of each pair of genes. The edge weight (*W*_*ij*_) is the importance of gene i in the tree model predicting gene j, which can be interpreted as the fraction of variance of the expression of gene j that can be explained by gene i^60^. However, the GENIE3 package is slow especially when it is deployed on genome-wide studies with a large number of experiments. So, to facilitate usage and improve speed, we implemented this algorithm by allowing users to define their regulators and by supporting parallel computing (Supplementary Fig. 7). In addition, in this random forest-based method we found the expressions of some genes were hardly predicted by other genes. So, we modified this algorithm by adding a filtering procedure to remove the poor random forest models (Supplementary Fig. 7). In other words, this procedure removed the genes and corresponding edges from the final network, whose expression was hardly predicted by the expression of the predefined regulators.

We provide users with an option to either supply a list of regulators of their interest or use the default list of regulators provided in *RegEnrich*, which were retrieved from three studies^61–63^. Using either COEN or GRN network, we then extract the regulator-target network by retaining the top-ranked edges (default = top 5% edges) between the regulators and their targets and subsequently filtering out nonconnected nodes. Apart from the data-driven network, *RegEnrich* also allows users to provide their regulator-target network, which can be derived from the literature, databases, or defined by the user using their data of other types.

### Enrichment analysis

The regulators are considered key regulators if they are differentially expressed along with their targets in a differentially expressed gene set. In other words, not only these regulator genes but also their target genes are differentially expressed upon different conditions. Finding these key regulators is an enrichment task, which is similar to retrieving the most overrepresented (enriched) biological annotations, such as gene ontology and pathways terms, of a list of interesting genes. Presently, *RegEnrich* provides users two options: Fisher’s exact test (FET) and gene set enrichment analysis (GSEA).

Fisher’s exact test (FET), also known as the hypergeometric test, calculates probability using the hypergeometric distribution (Eq. 1). This distribution describes the probability of the number of draws being successful (*k*) within a sequence of draws (*M*), without replacement, from a finite population (*N*) consisting of two types of elements (the total number of successful types is *s*).

1 $$p\left(k{{{{{\rm{;}}}}}}s,M,N\right)=\frac{\left(\begin{array}{c}M\\ k\end{array}\right)\left(\begin{array}{c}N-M\\ s-k\end{array}\right)}{\left(\begin{array}{c}N\\ s\end{array}\right)}$$

Then the *p* value, depicting the probability of observing *K* (and more) differential targets by chance, of regulator *i* being overrepresented, is calculated by

2 $${{{{{{\rm{P}}}}}}}_{i}=\mathop{\sum }\limits_{k=K}^{{S}_{i}}{p}_{i}\left(k{{{{{\rm{;}}}}}}{s}_{i},M,N\right)=\mathop{\sum }\limits_{k=K}^{{S}_{i}}\frac{\left(\begin{array}{c}M\\ k\end{array}\right)\left(\begin{array}{c}N-M\\ {s}_{i}-k\end{array}\right)}{\left(\begin{array}{c}N\\ {s}_{i}\end{array}\right)}$$

where *N* is the total number of genes in the previously constructed regulator-target network; M is the number of genes in the list of users’ interests (the genes not in the network are excluded), which is typically the differential genes between conditions; ${s}_{i}$ is the number of target genes of regulator *i* in the network; *K* is the number of target genes that are also in the list of users’ interests. This process repeats for all regulators that are predefined by users.

Gene set enrichment analysis (GSEA) is one of the most widely used methods to study the biological function of groups of genes and to interpret gene expression data^64^. GSEA takes into account all of the genes in an experiment, unlike FET that takes into account only those genes above a fold-change or significance cutoff. Here, *RegEnrich* takes two basic inputs, the TF-target network and a named vector of decreasingly sorted ranking metrics (*r, z*-score scaled negative logarithm of differential significance p-values) of all genes. Briefly, there are three major steps in this analysis:

1. Calculation of an Enrichment Score (ES) by:
  3 $${{{{{\rm{ES}}}}}}=\left\{\begin{array}{c}{{\max }}\left(\Delta P\right),{{\max }}\left(\Delta P\right)\ge {{\max }}\left(-\Delta P\right)\hfill\\ {{\min }}\left(\Delta P\right), {{\max }}\left(\Delta P\right) \, < \, {{\max }}\left(-\Delta P\right)\end{array}\right.,$$
  where
  4 $$\Delta {P}_{i}={P}_{{hit}}(S,i)-{P}_{{miss}}(S,i),$$
  where
  5 $${P}_{{hit}}\left(S,i\right)=\mathop{\sum}\limits _{{g}_{j}\in S,j\le i}\frac{\left|{r}_{j}\right|}{\mathop{\sum }\limits_{{g}_{j}\in S}\left|{r}_{j}\right|},{P}_{{miss}}(S,i)=\mathop{\sum}\limits_{{g}_{j}\notin S,j\le i}\frac{1}{(N-{N}_{H})}$$
  Here, *i* is the index of decreasingly sorted ranking metrics *r*, *S* is the set of target genes of one particular regulator, *N*_*H*_ is the number of genes in *S*, and *N* is the total number of valid genes in the regulator-target network.
2. Randomly shuffle the ranking metrics of genes and re-compute ES. And repeat this process for 1000 permutations to generate ES_NULL_ that establishes an empirical distribution. Estimate empirical *p* value for S from ES_NULL_ by only the positive portion of the distribution corresponding to the sign of the observed ES.
3. Perform steps 1 and 2 for each regulator, generating a numeric vector (${P}_{E}$) in which each value is an enrichment *p* value for each regulator.

### Regulator ranking and visualization

After the enrichment analysis by either FET or GSEA, the overall ranking scores of regulators were calculated by:

6 $${score}=f(-{\log }({P}_{E}))+f(-{\log }({P}_{D}))$$

where $f(x)=\frac{x-{\min }\left(x\right)}{{\max }\left(x\right)-{\min }\left(x\right)}$, and ${P}_{D}$ is the vector of *p* values of regulators obtained from differential expression analysis, ${P}_{E}$ is the vector of p-values of regulators obtained from the enrichment analysis.

In the *RegEnrich* package, we have implemented several functions for visualizing the information of a regulator and its targets (Fig. 1). For example, “plotRegTarExpr” function is to plot the expression pattern of a regulator and its targets.

### Master regulator inference analysis based on ARACNe + viper algorithm

According to the official tutorial on GitHub (https://github.com/califano-lab/ARACNe-AP), we reverse-engineered the regulatory networks using the gene expression datasets obtained from either GEO database or the simulated dataset, based on the ARACNe-AP package with default parameters except the TF list. To make the results comparable between viper and RegEnrich, here, the TF list is set as the same as the default regulators in *RegEnirch*. The regulon object is generated from the ARACNe network file and the corresponding expression dataset using the aracne2regulon function from the viper package with default parameters. Either the paired t-test or t-test was applied to compare the gene expression change between groups, depending on whether the experiment is a paired study. Meanwhile the sample permutation and paired *t*-test or *t*-test were used to generate a null model. The t-statistics and corresponding p-values, and the null model were used to perform master regulator inference analysis with msviper function from viper package. To confirm the results by using the network that we built, we reanalyzed the gene-silencing datasets using the public network of corresponding cancer type from the aracne.networks package (version 1.16.0).

### Statistics and reproducibility

*T*-test was used to compare the gene expression changes between groups to obtain master regulon by VIPER when the samples between groups are not paired, while paired T-test was used when the samples between groups are paired. To perform the enrichment analysis in the third step of RegEnrich pipeline, either GSEA method or Fisher’s exact test was used. To compare the performance of *RegEnrich* and the VIPER package, the ranks of key/master regulators identified by both methods using the simulated gene expression data were compared using the GSEA method. To evaluate the influence of the sample size on the performance of *RegEnrich* and VIPER package, the maximum sample size is set to 100 and the sample size is reduced to 50, 20, 10, and 6 by down-sampling strategy. The datasets of gene knock-down experiments and IFN stimulation experiments with sample sizes in each condition ≥ 3 were used for evaluation. All of the statistical analyses were performed using the R software (version 4.01).

### Reporting summary

Further information on research design is available in the Nature Research Reporting Summary linked to this article.

## Supplementary information

Supplementary Information

Description of Additional Supplementary Files

Supplementary Data 1

Supplementary Data 2

Reporting Summary

## Data Availability

All of the gene expression data were downloaded from GEO database according to the GEO accession ID listed in the Tables 1 and 2. The source data underlying Fig. 2 are provided in Supplementary Data 1. The source data underlying Figs. 3 and 4 are provided as Supplementary Data 2. Any other relevant data are available upon reasonable request.

## Code availability

The source codes of RegEnrich package are publicly available on Bioconductor (https://www.bioconductor.org/packages/release/bioc/html/RegEnrich.html). The source codes used for analyzing the public data and the simulated data are available on GitHub (https://github.com/paodan/RegEnrich_CustomCode).
