# Supplementary material for: RegEnrich gene regulator enrichment analysis reveals a key role of the ETS transcription factor family in interferon signaling
Source: Commun Biol. 2022 Jan 11;5:31. doi: 10.1038/s42003-021-02991-5 (PMC8752721; doi:10.1038/s42003-021-02991-5)
Supplement: Supplementary file 2 — Description of Additional Supplementary Files [file 42003_2021_2991_MOESM2_ESM.pdf]

Description of Additional Supplementary Files

File name: Supplementary Data 1

Description: Memory usage and time consumption by RegEnrich analyzing different sizes of data.

File name: Supplementary Data 2

Description: Source data underlying Figure 3 and 4.
